# Fracture Resistance and Stress Distribution of Weakened Bovine Roots Restored with Different Glass Fiber Post Systems

**DOI:** 10.3390/dj14100651

**Published:** 2026-10-08

**Authors:** Felipe Bernardo de Moura, Guilherme Andrade, Alexandre Borges, Cláudio Carvalho

**Affiliations:** 1 Department of Restorative Dentistry, Endodontics Division, Institute of Science and Technology, São Paulo State University (UNESP), São José dos Campos 12245-000, SP, Brazil; 2 Department of Dental Materials and Prosthodontics, Institute of Science and Technology, São Paulo State University (UNESP), São José dos Campos 12245-000, SP, Brazil; guisdandrade@hotmail.com (G.A.); alexandre.borges@unesp.br (A.B.)

**Keywords:** glass fiber posts, fracture resistance, weakened roots, endodontically treated teeth, finite element analysis, Splendor-SAP

## Abstract

**Background/Objectives:** The restoration of structurally weakened roots remains a clinical challenge. This in vitro study evaluated the fracture resistance, failure pattern, and stress distribution of weakened bovine roots restored with two glass fiber post systems under different remaining root dentin thicknesses. **Methods:** Sixty bovine incisor roots were divided into six groups (*n* = 10): G1, 2 mm of remaining root dentin without a post; G2, 3 mm without a post; G3, 2 mm with an Exacto post; G4, 3 mm with an Exacto post; G5, 2 mm with a Splendor-SAP post; and G6, 3 mm with a Splendor-SAP post. Specimens underwent root canal treatment and standardized restoration, followed by 1,200,000 mechanical cycles at 88 N and 3.8 Hz and fracture testing at 45°. Finite element models were subjected to an 88 N oblique load. Fracture resistance was analyzed using Welch’s one-way ANOVA followed by Games–Howell post hoc comparisons, whereas failure patterns were analyzed using the Fisher–Freeman–Halton exact test. **Results:** Fracture resistance differed significantly among groups (*p* < 0.001). G1 (88.7 N) and G2 (95.6 N) showed the lowest and statistically similar values; G3 (670.9 N) and G5 (691.9 N) showed intermediate and statistically similar values; and G4 (786.2 N) and G6 (784.8 N) showed the highest and statistically similar values. Failure-pattern distribution was not significantly associated with the experimental groups (*p* = 0.582). Finite element analysis showed stress concentration predominantly in the cervical region of the root, with G5 showing the highest maximum principal stress in root dentin. **Conclusions:** Glass fiber posts substantially increased the fracture resistance of weakened roots. Among post-restored specimens, roots with 3 mm of remaining dentin showed higher fracture resistance than those with 2 mm, whereas no significant difference was observed between the Exacto and Splendor-SAP systems under the same remaining-dentin condition.

## 1. Introduction

Extensive loss of dental structure resulting from carious lesions, large restorations, trauma, or alterations in root development represents an important challenge in restorative dentistry [1,2,3]. In such cases, tooth preservation depends on the quantity and quality of the remaining dental structure, as well as on the selection of an appropriate restorative strategy. In teeth with weakened roots, reduced dentin thickness may compromise mechanical resistance and increase susceptibility to cracks and root fractures [4,5,6].

Endodontically treated teeth may present substantial structural compromise, particularly when associated with significant loss of coronal and radicular dentin [1,2]. Importantly, endodontic treatment itself does not appear to make dentin intrinsically more brittle; rather, loss of tooth structure is considered a major contributor to the mechanical compromise of endodontically treated teeth [7]. This condition is particularly critical in immature teeth and in teeth with enlarged root canals or weakened roots, in which the amount of remaining dentin is reduced [4,5,6,8,9,10].

The restoration of endodontically treated teeth with extensive coronal structural loss may require an intraradicular post to provide retention for the core and coronal restoration [1,2]. Several post systems are available and differ in material, geometry, elastic modulus, and clinical technique [11,12,13]. Cast metal posts and cores have been widely used; however, their greater stiffness may promote unfavorable stress distribution within the remaining root structure and increase the risk of irreparable fractures [14,15,16,17]. In addition, metallic post systems may present esthetic limitations, particularly in anterior restorations [11,12,13].

In this context, glass fiber posts have been widely used because their elastic modulus is closer to that of dentin than that of metallic post systems. Representative values reported in the finite element literature relevant to the present study are approximately 18.6 GPa for dentin and 31.5 GPa for glass fiber posts [4]. This relative similarity in stiffness may favor more homogeneous stress transfer within the restored tooth, while glass fiber posts also provide favorable esthetic properties [18,19]. Nevertheless, the role of intraradicular posts in strengthening endodontically treated teeth remains debated. Although some experimental studies have reported increased fracture resistance after post placement, the primary purpose of a post is to provide retention for the core and coronal restoration rather than to intrinsically reinforce the remaining root structure [20,21].

The thickness of the remaining root dentin is an important factor in the prognosis of structurally weakened teeth. In roots with thin dentinal walls, additional preparation for post adaptation may further reduce the remaining structure and potentially compromise the restored tooth [22,23]. Therefore, post systems that can accommodate enlarged root canals while minimizing additional dentin removal are of particular interest. The Splendor-SAP system (Angelus, Londrina, PR, Brazil) consists of an adjustable glass fiber post associated with a sleeve, a configuration intended to improve adaptation within enlarged canals and reduce the volume of resin cement required for post cementation [24,25,26]. However, whether this design influences fracture resistance and stress distribution in weakened roots with different amounts of remaining dentin requires further investigation.

Finite element analysis has been used as a complementary method to investigate the biomechanical behavior of restored teeth. This approach enables standardized simulation of different structural and restorative conditions and allows identification of regions of stress concentration within the modeled structures. Accordingly, combining fracture resistance testing with finite element analysis may provide complementary information on the mechanical behavior of weakened roots restored with different glass fiber post systems and different amounts of remaining dentin [4,27,28].

Therefore, the aim of this in vitro study was to evaluate the fracture resistance, failure pattern, and stress distribution of weakened bovine roots restored with the Exacto (Angelus, Londrina, PR, Brazil) and Splendor-SAP glass fiber post systems under two standardized conditions of remaining root dentin thickness. By combining mechanical testing and finite element analysis, the study sought to determine whether the amount of remaining dentin and the configuration of the glass fiber post system influence the mechanical behavior of structurally weakened roots.

The null hypotheses were that (i) the experimental conditions defined by the glass fiber post system and remaining root dentin thickness would not influence fracture resistance or failure pattern and (ii) the different experimental configurations would not affect stress distribution in the finite element models.

## 2. Materials and Methods

### 2.1. Experimental Design

This in vitro study evaluated fracture resistance, failure pattern, and stress distribution in weakened bovine roots under six experimental conditions defined by remaining root dentin thickness (2 or 3 mm) and glass fiber post system (none, Exacto, or Splendor-SAP). Sixty sound bovine incisor roots were included.

An a priori sample size calculation was not performed. The sample size of 10 specimens per group was established based on previous in vitro studies using bovine incisors and comparable fracture-resistance methodologies [4,10].

After specimen standardization according to the eligibility criteria, the roots were sequentially allocated to the six experimental groups. The first specimen was assigned to G1, the second to G2, and so forth through G6; this sequence was repeated until each group comprised 10 specimens. The groups were defined according to cervical remaining root dentin thickness and the glass fiber post system used.

The distribution of the experimental groups is presented in Table 1.

### 2.2. Specimen Selection and Preparation

Sound bovine incisors with completely closed root apices were selected. The teeth were cleaned by root scaling with periodontal curettes, stored in sealed containers containing saline solution, and kept frozen until use. Information regarding animal age was not available.

Only teeth with straight roots and a cervical root diameter of 10 ± 0.5 mm, measured using a digital caliper, were included. The crowns were sectioned below the cementoenamel junction using a carborundum disc (Dentorium, São Paulo, SP, Brazil), standardizing the root length to 16 mm. All specimens were prepared by a single operator.

Pulp tissue was removed using #45 Hedström files (Dentsply/Maillefer, Ballaigues, Switzerland). The root canals were manually instrumented to a standardized final instrument size of #80 using K-files (Maillefer-Dentsply, Petrópolis, RJ, Brazil). This fixed final instrument size was used to standardize the initial canal preparation before the controlled root-weakening procedure; therefore, the instrumentation protocol was not intended to reproduce a contemporary clinical instrumentation technique or to constitute an experimental variable.

During biomechanical preparation, the canals were irrigated with 2.5% sodium hypochlorite solution (Soda Clorada–Asper, Santa Maria, São Caetano do Sul, SP, Brazil), totaling 10 mL per specimen.

### 2.3. Simulation of Root Weakening

Root weakening was performed in the cervical and middle thirds of the canals to reproduce two standardized degrees of structural loss. Remaining root dentin thicknesses of 2 and 3 mm represented, respectively, a more severely weakened condition and a condition with a greater amount of remaining radicular dentin. These dimensions were used as standardized experimental conditions rather than as clinical threshold values [5,6].

Initially, the canals of all specimens were enlarged to a depth of 12 mm using Gates-Glidden burs (AR Maillefer-Dentsply, Petrópolis, RJ, Brazil) sizes #3, #4, #5, and #6. During this procedure, the roots were stabilized in a bench vise to minimize specimen movement.

Subsequently, the specimens were fixed to the support of a circular specimen-cutting device, allowing the burs to be inserted parallel to the long axis of the root canal. The internal dentin walls were reduced under distilled-water irrigation. A #720 round-ended tapered diamond bur (Microdont, Monsey, NY, USA) at low speed was used in the first 6 mm of the root canal. A #707G flat-ended tapered bur (KG Sorensen, Cotia, SP, Brazil), also at low speed, was then used to enlarge the canal up to 12 mm in depth, maintaining 4 mm of apical dentin without preparation.

Preparation was continued until cervical remaining root dentin thicknesses of 2 or 3 mm were obtained, according to the experimental group.

Root-wall thickness was confirmed by digital radiographs (NanoPix–Eighteeth, Changzhou, China) obtained in the buccolingual and mesiodistal directions. The roots were positioned on the digital radiographic sensor with the aid of utility wax. A radiographic positioning device was used to standardize image acquisition, maintaining the X-ray beam perpendicular to the root/sensor assembly and a standardized distance of 10 cm. Radiographs were obtained using an intraoral X-ray unit (Kodak 2200, Intraoral X-ray System), and root-wall thickness was measured using the measurement tools of the NanoPix software (version 1.1.1.8).

After radiographic confirmation of root-wall thickness, the specimens were examined under a stereomicroscope at 7.5× magnification (Discovery V20–Zeiss, Munich, Germany) to verify the absence of cracks or structural defects that could interfere with the mechanical resistance of the roots.

**Figure 1:**
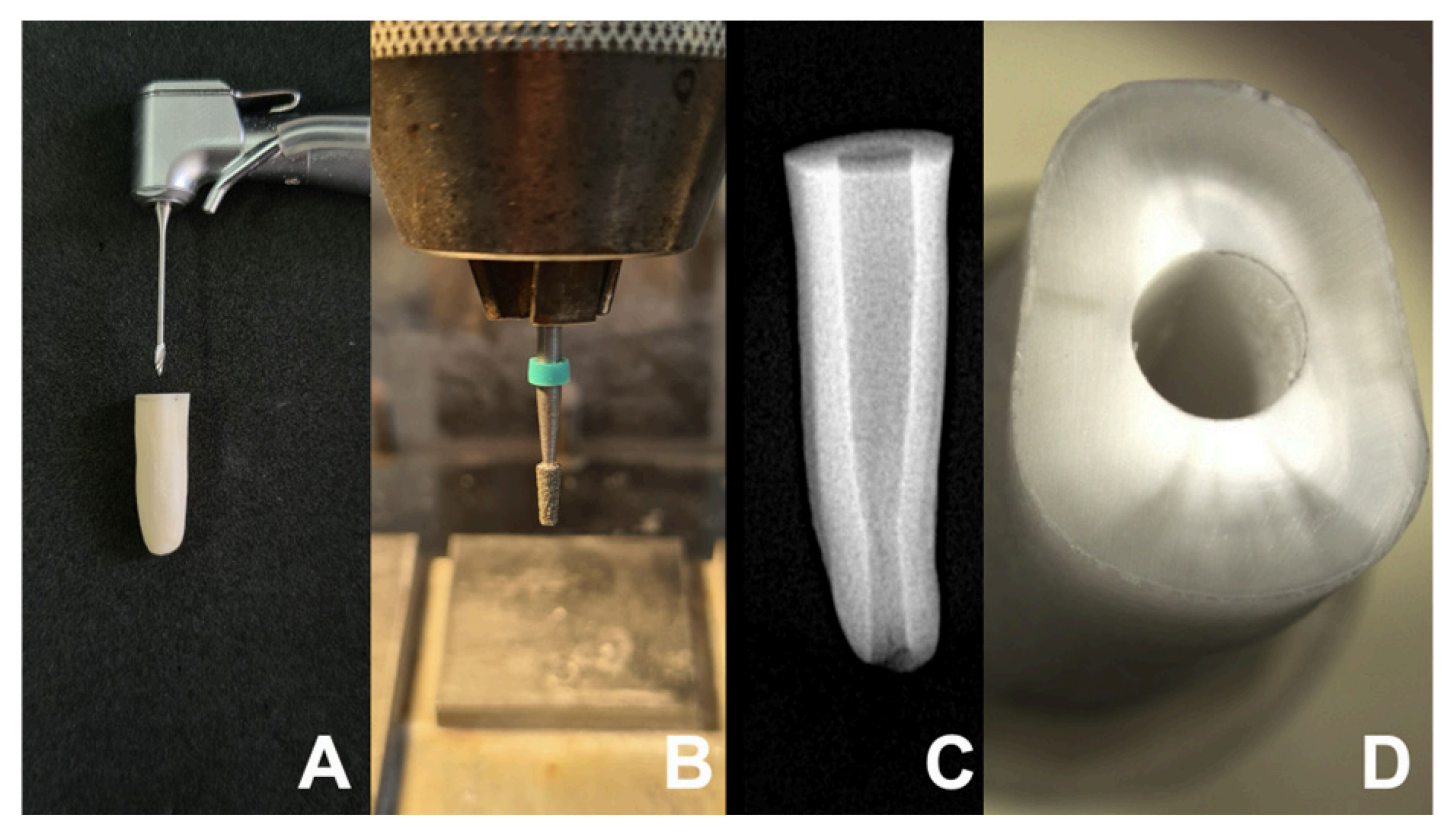
Root weakening and specimen inspection: (**A**) preparation with a #4 Gates-Glidden bur; (**B**) #707G flat-ended tapered bur fixed in the circular specimen-cutting device; (**C**) radiographic confirmation; and (**D**) stereomicroscopic analysis.

The root-weakening and specimen-inspection procedures are illustrated in Figure 1.

### 2.4. Root Canal Filling

The root canals of all specimens were filled along their entire length with gutta-percha cones (Dentsply, Petrópolis, RJ, Brazil), an epoxy resin-based endodontic sealer (AH Plus, Dentsply, Petrópolis, RJ, Brazil), and the active lateral condensation technique to standardize all specimens.

After root canal filling, 12 mm of the filling material was removed from the cervical and middle thirds using electric pluggers (Eighteeth, Changzhou, China), leaving 4 mm of filling material in the apical third. Vertical condensation of the remaining filling material was then performed using a plugger (Golgran, São Caetano do Sul, SP, Brazil).

The internal walls of the canals were cleaned with ethyl alcohol (Itajá–Jalles Machado S/A, Goianésia, GO, Brazil), and the cavities were temporarily sealed with a zinc oxide-based cement (Vigodent–Coltene, Brazil). The specimens were stored for 72 h in a 100% humid environment to allow complete setting of the endodontic sealer.

### 2.5. Specimen Embedding and Periodontal Ligament Simulation

A standardized space for periodontal ligament simulation was created by immersing the roots in melted utility wax for 5 s, producing an approximately 0.2 mm thick wax spacer around the root surface. The wax served exclusively as a spacer to create the space subsequently occupied by the periodontal ligament-simulating material.

The roots were positioned in PVC cylinders measuring 25 mm in diameter and 30 mm in height, which were filled with chemically activated acrylic resin (JET, Artigos Odontológicos Clássicos, São Paulo, SP, Brazil). Positioning was performed with the aid of a dental surveyor, maintaining 2 mm of the coronal portion of the root exposed above the resin block.

After polymerization of the acrylic resin, the roots were removed from the cylinders and the wax layer was completely eliminated. The resulting space between the root and the acrylic resin was filled with condensation silicone (3M, St. Paul, MN, USA), and the roots were repositioned. This procedure produced an approximately 0.2 mm thick resilient silicone layer surrounding the root surface to simulate the periodontal ligament, following approaches previously described in laboratory studies [5,10].

**Figure 2:**
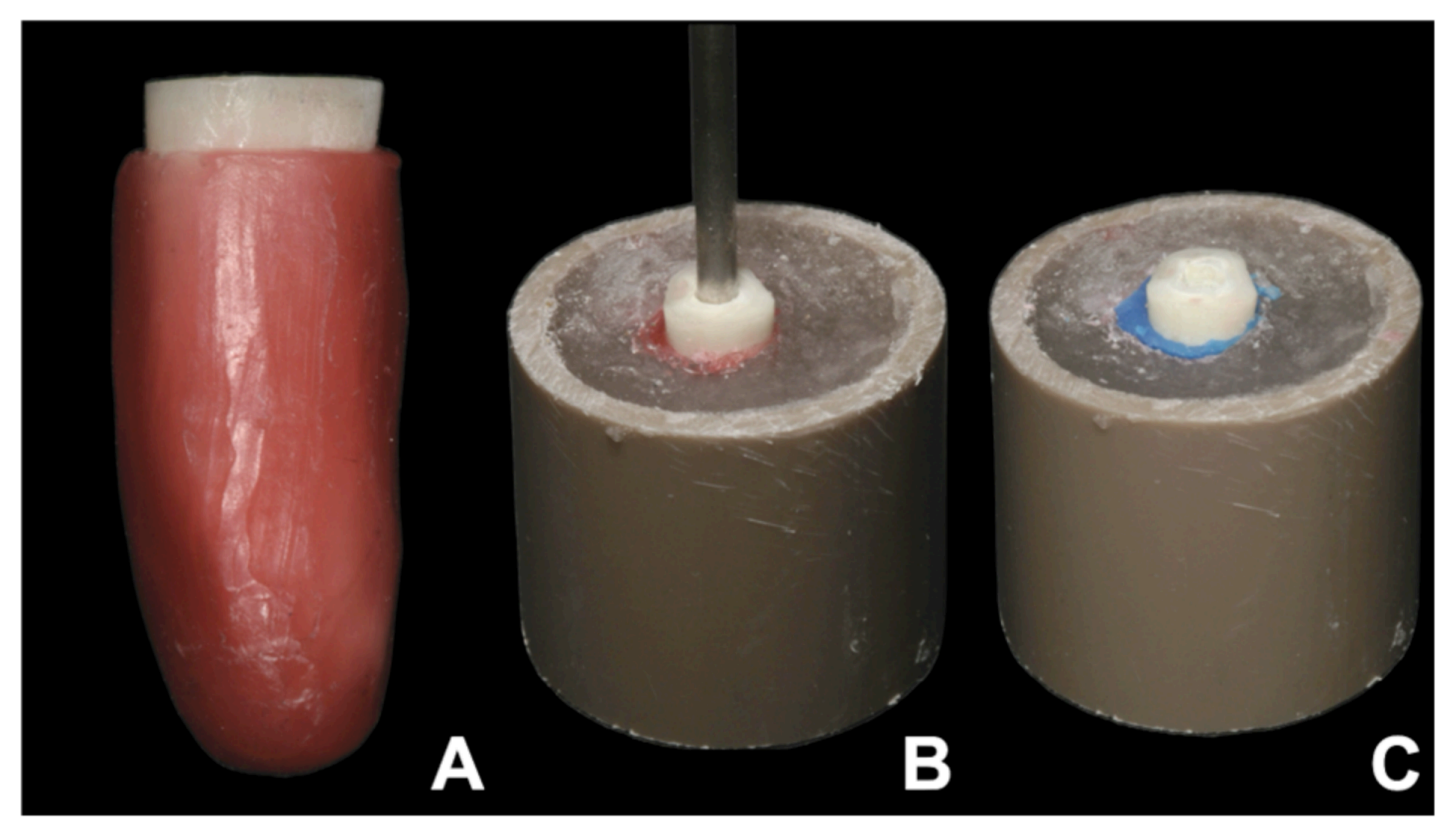
Specimen embedding and periodontal ligament simulation: (**A**) specimen covered with a thin wax layer; (**B**) specimen after embedding in acrylic resin; and (**C**) periodontal ligament simulation using silicone.

The embedding and periodontal ligament simulation procedures are shown in Figure 2.

### 2.6. Intraradicular Restoration

The Exacto and Splendor-SAP systems were selected because they represent two different glass fiber post configurations for the restoration of enlarged root canals. Exacto is a conventional prefabricated glass fiber post, whereas Splendor-SAP incorporates an adjustable post-and-sleeve configuration. Their comparison allowed the mechanical behavior of two post configurations to be evaluated under the same standardized degrees of root weakening.

Specimens from groups G1 and G2 did not receive an intraradicular post.

In groups G3 and G4, an Exacto glass fiber post (Angelus, Londrina, PR, Brazil) was used. Initially, the posts were cleaned with alcohol (Itajá–Jalles Machado S/A, Goianésia, GO, Brazil), positioned inside the root canals, and radiographs were obtained to verify post adaptation and positioning. After radiographic confirmation, the canals were prepared according to the manufacturer’s instructions.

The canals were etched with 37% phosphoric acid FUSION-DURALINK (Angelus, Londrina, PR, Brazil) for 30 s and dried with absorbent paper points (Tanari, Manacapuru, AM, Brazil). The posts were cleaned with alcohol, air-dried, and treated with silane (Angelus, Londrina, PR, Brazil) for 60 s, followed by air-drying. A dentin adhesive system (Ambar, FGM, Joinville, SC, Brazil) was applied inside the root canals and, after 20 s, the canals were air-dried.

Dual-cure resin cement (Allcem Core, FGM, Joinville, SC, Brazil) was inserted into the canal in an apical-to-cervical direction according to the manufacturer’s recommendations. The Exacto posts were positioned inside the canals, excess cement was removed, and light curing was performed for 40 s using an LED curing unit (3M^TM^ Elipar^TM^ DeepCure-L).

In groups G5 and G6, the Splendor-SAP system (Angelus, Londrina, PR, Brazil) was used. The post and sleeve were cleaned with alcohol, positioned inside the root canal, and evaluated radiographically to confirm adaptation and positioning.

The canals were etched with FUSION-DURALINK for 30 s and dried with paper points. The post and sleeve received silane application for 60 s, followed by air drying. The dentin adhesive was applied inside the canals and, after 20 s, the canals were air-dried. Dual-cure resin cement was inserted into the canal in an apical-to-cervical direction, and the Splendor-SAP assembly was positioned according to the manufacturer’s recommendations. Excess cement was removed, and light curing was performed for 40 s.

**Figure 3:**
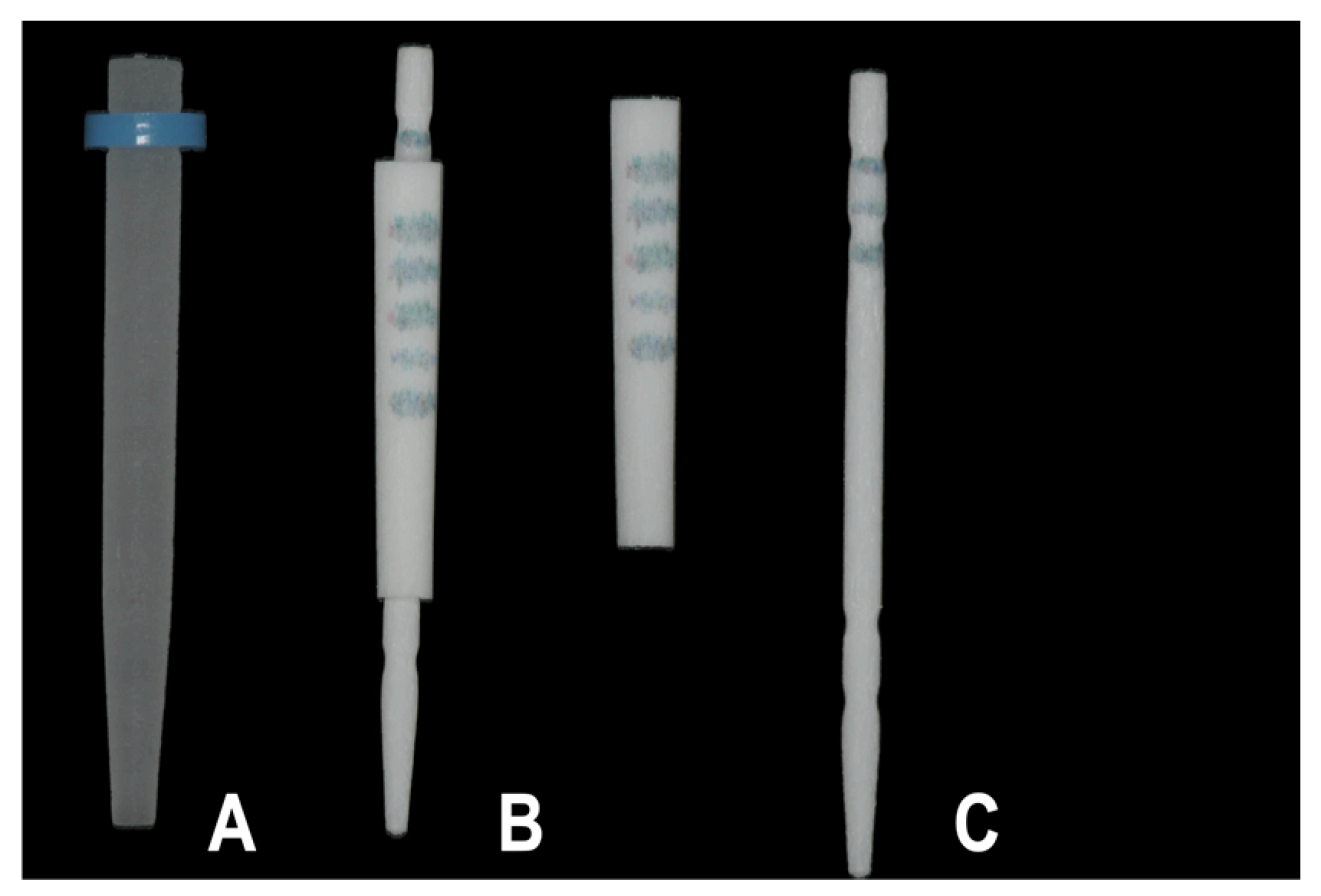
Glass fiber post systems: (**A**) Exacto 3 glass fiber post; (**B**) Splendor-SAP glass fiber post system; and (**C**) separated Splendor-SAP components, showing the post and sleeve.

The glass fiber post systems used in the study are shown in Figure 3.

### 2.7. Coronal Reconstruction

**Figure 4:**
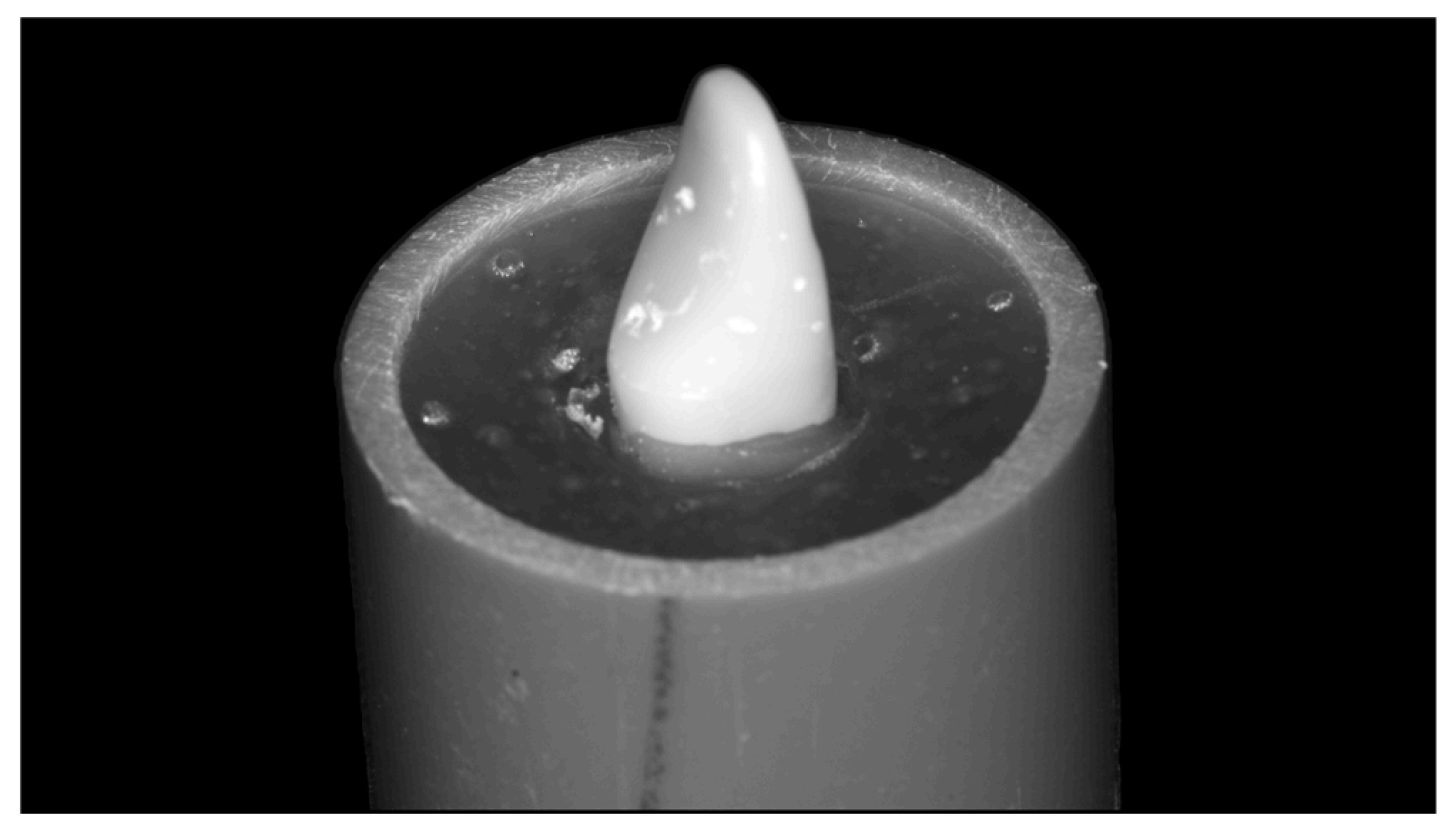
Specimen with the periodontal ligament simulated using silicone and a standardized reconstructed crown.

To standardize the coronal portion of the specimens, previously fabricated acetate matrices were used. These matrices reproduced the shape of a natural central incisor prosthetic abutment, as shown in Figure 4. These matrices were obtained from wax-sculpted crowns that were molded and replicated to allow standardized coronal reconstruction.

The matrices were adapted to the roots and filled with dual-cure resin cement, forming the standardized coronal reconstruction of the specimens.

A specimen after standardized coronal reconstruction is shown in Figure 4.

### 2.8. Mechanical Cycling

The specimens were subjected exclusively to mechanical cycling in a thermomechanical cycling machine (ER-37000, ERIOS, São Paulo, SP, Brazil). Although the equipment was capable of thermal cycling, no thermocycling was performed in the present study.

The mechanical cycling protocol consisted of 1,200,000 cycles under an 88 N load at a frequency of 3.8 Hz. These parameters were selected based on previously described fatigue protocols using weakened bovine roots and comparable glass fiber post restorations [29,30].

Load application was performed using a spherical-tip piston measuring 4 mm in diameter, positioned on the lingual surface of the reconstructed crowns, 3 mm from the incisal edge.

**Figure 5:**
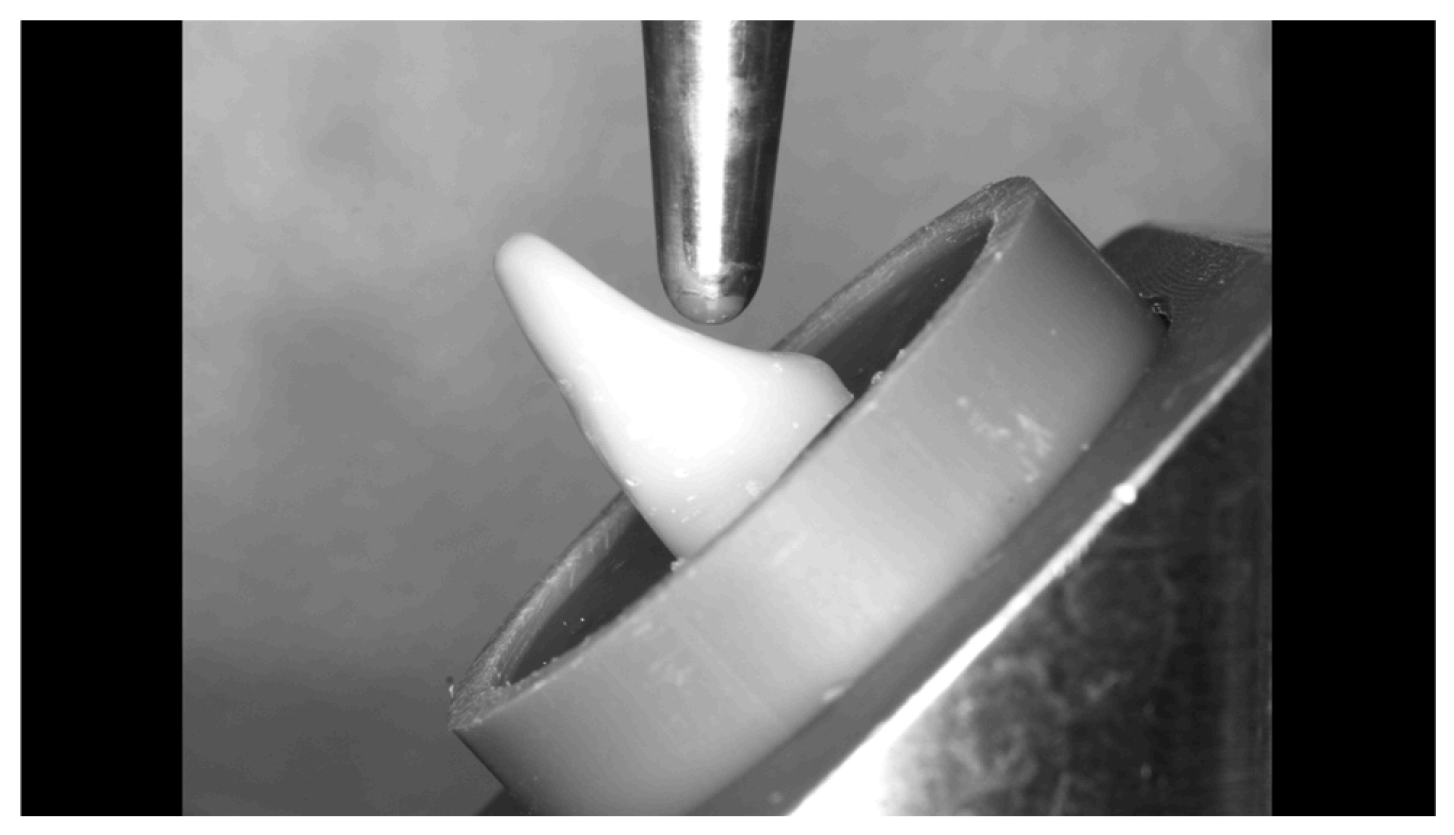
Spherical-tip piston positioned on the lingual surface of the reconstructed specimen crown.

The loading piston position is shown in Figure 5.

### 2.9. Fracture Resistance Test

After mechanical cycling, the specimens were subjected to a fracture resistance test using a universal testing machine (DL-1000, EMIC, São José dos Pinhais, PR, Brazil). The specimens were fixed in a cylindrical stainless-steel device that allowed positioning at a 45° inclination relative to the direction of load application.

The test was performed using a 100 kgf (981 N) load cell at a crosshead speed of 1 mm/min until specimen fracture. The maximum load supported by each specimen was recorded in newtons and subsequently tabulated for statistical analysis.

Before the fracture resistance test, the silicone layer used to simulate the periodontal ligament was removed, and the specimens were re-embedded in acrylic resin. This procedure was performed to prevent movement of the root–crown assembly during mechanical testing.

**Figure 6:**
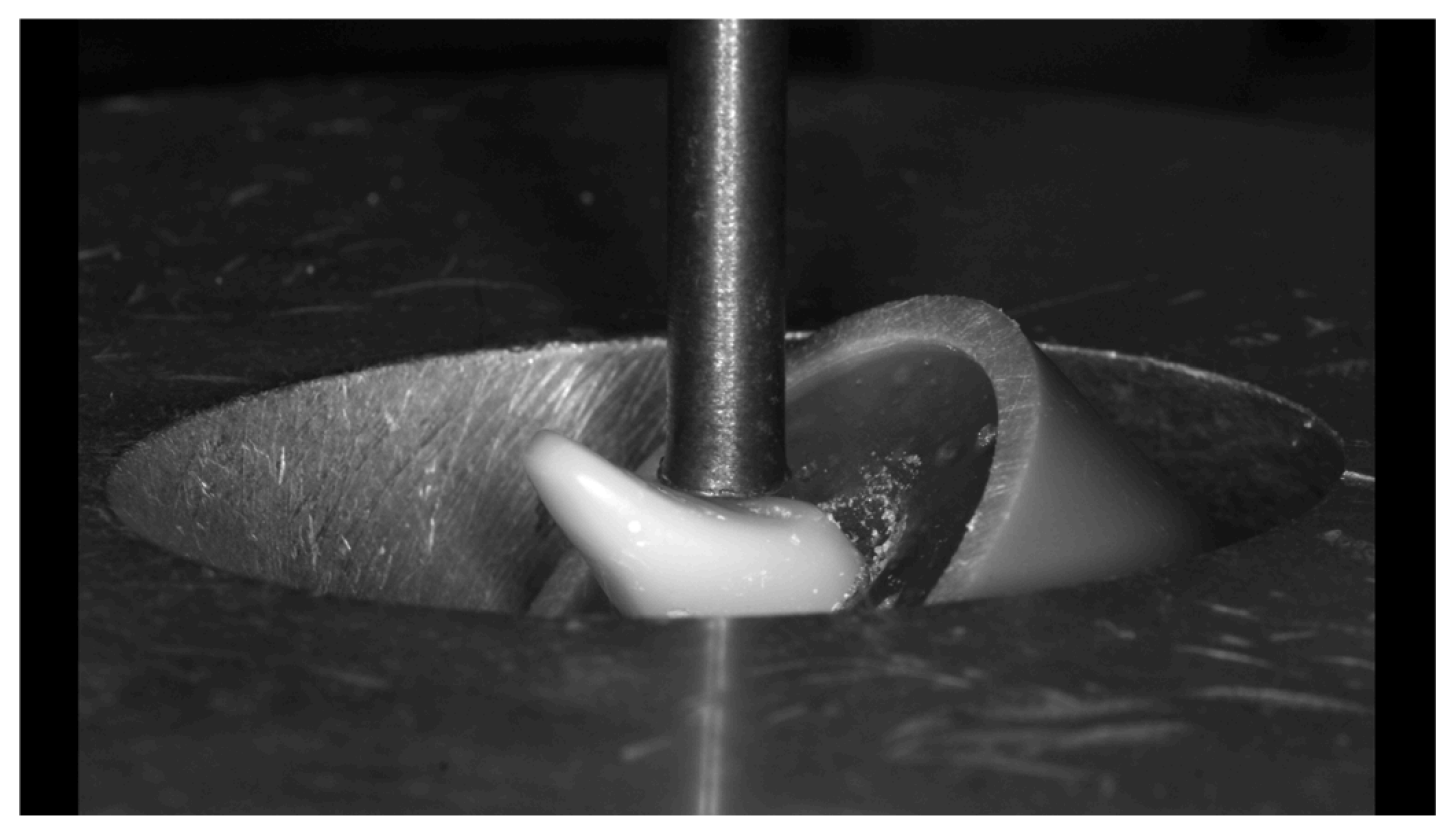
Specimen positioned at a 45° inclination for the compression test.

The specimen position during the compression test is shown in Figure 6.

### 2.10. Failure Pattern Analysis

After the fracture resistance test, the specimens were examined under a stereomicroscope at 7.5× magnification to classify the failure mode.

Failures were classified into three types:

- Type I: fracture or displacement of the crown and/or post above the simulated bone crest, allowing preservation of the remaining root structure.
- Type II: fracture located in the cervical third of the root, also allowing preservation of the remaining root structure.
- Type III: catastrophic fracture, characterized by vertical or oblique fractures extending below the simulated bone crest, preventing preservation of the remaining root structure.

**Figure 7:**
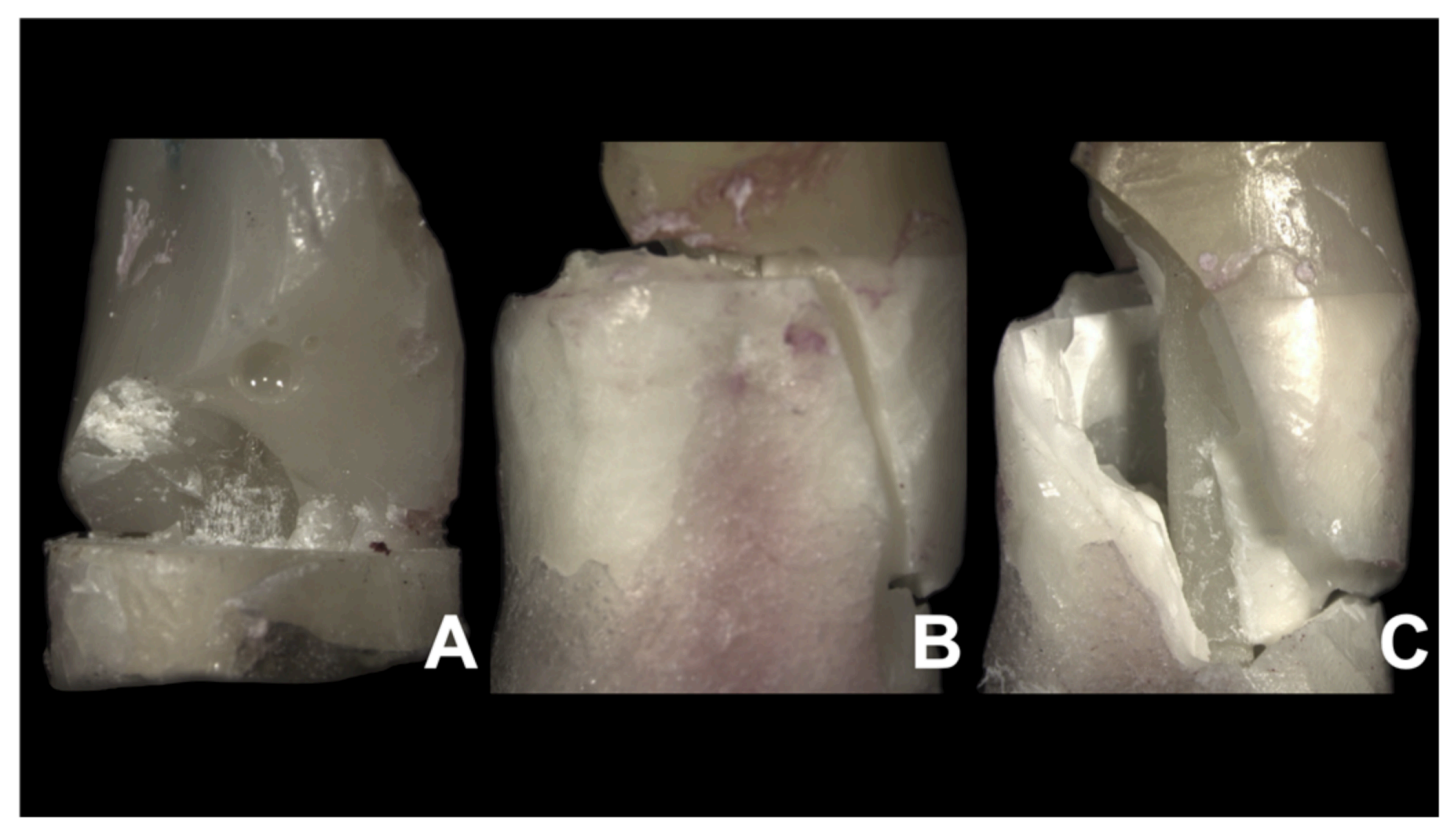
Representative failure patterns: (**A**) type I fracture; (**B**) type II fracture; and (**C**) type III fracture.

Representative failure patterns are shown in Figure 7.

### 2.11. Finite Element Analysis

Finite element analysis was performed to evaluate the biomechanical behavior and stress distribution in weakened bovine roots with and without intraradicular posts. Three-dimensional models representative of the six experimental groups were constructed, reproducing the dimensions of the specimens used in the mechanical test, including the remaining root dentin, simulated internal canal weakening, apical filling material, resin cement, glass fiber post systems, and coronal reconstruction.

Three-dimensional modeling was performed using Rhinoceros 6.0 SR8 software (Robert McNeel & Associates, Seattle, WA, USA), following methodologies previously described for dental models subjected to finite element analysis. The models were subsequently imported into ANSYS 19.2 software (ANSYS Inc., Canonsburg, PA, USA) for structural analysis [4,27,28].

The mesh was generated using tetrahedral elements, totaling 316,768 nodes and 206,340 elements. A 10% mesh convergence test was performed to ensure that mesh refinement did not interfere with the results. The mechanical properties of the materials were assigned based on the literature, considering isotropic, homogeneous, linear, and elastic behavior.

All interfaces between model components were defined as bonded contacts, representing an idealized condition of complete adhesion without interfacial sliding or separation. This assumption was adopted to allow standardized comparison of the effects of remaining dentin thickness and post-system configuration without introducing interfacial debonding as an additional variable.

The model was fixed at the base of the support cylinder, and an oblique load of 88 N was applied to the lingual region of the coronal reconstruction at a 45° angle to the long axis of the root. This loading condition reproduced that used during mechanical cycling and allowed standardized comparison of stress distribution among the experimental groups.

Colorimetric maps of von Mises equivalent stress, maximum principal stress in root dentin, maximum principal stress in the post-and-core build-up, and total deformation were obtained. Maximum principal stress was used as the main criterion for evaluating tensile stress in radicular dentin.

The material properties assigned to the finite element models are presented in Table 2.

### 2.12. Statistical Analysis

Fracture resistance data were analyzed at a significance level of 5%. Data normality was assessed using the Shapiro–Wilk test, and homogeneity of variances was evaluated using Levene’s test.

Because heterogeneity of variances was detected, differences in fracture resistance among the experimental groups were primarily analyzed using Welch’s one-way analysis of variance, followed by Games–Howell post hoc comparisons.

A conventional one-way analysis of variance followed by Tukey’s honestly significant difference test was additionally performed as a sensitivity analysis to assess the robustness of the findings.

The magnitude of the overall group effect on fracture resistance was estimated using eta-squared (η^2^). Means, sample standard deviations, and 95% confidence intervals were calculated for each experimental group.

Failure-pattern data were treated as categorical variables. Because several expected cell frequencies were below five, the association between experimental group and failure pattern was assessed using the Fisher–Freeman–Halton exact test.

Statistical significance was set at α = 0.05. All statistical analyses were performed using GraphPad Prism, version 11 (GraphPad Software, LLC, Boston, MA, USA).

## 3. Results

### 3.1. Fracture Resistance

The mean fracture resistance values, sample standard deviations, 95% confidence intervals, and statistical groupings are presented in Table 3.

The Shapiro–Wilk test indicated no significant deviation from normality in any experimental group (*p* = 0.148–0.953). However, Levene’s test indicated heterogeneity of variances among groups (F(5,54) = 4.328; *p* = 0.002). Therefore, fracture resistance was primarily compared using Welch’s one-way analysis of variance.

Welch’s ANOVA demonstrated a significant difference in fracture resistance among the experimental groups (F(5,24.86) = 1162.11; *p* < 0.001), with a large overall effect size (η^2^ = 0.986). Games–Howell post hoc comparisons identified three statistically distinct groupings. G1 and G2 showed the lowest and statistically similar fracture resistance values. G3 and G5 showed intermediate and statistically similar values, whereas G4 and G6 showed the highest and statistically similar values.

A sensitivity analysis using conventional one-way ANOVA also showed a significant difference among groups (F(5,54) = 762.95; *p* < 0.001), and Tukey’s HSD test yielded the same pattern of pairwise differences as the Games–Howell analysis.

### 3.2. Failure Pattern

The distribution of failure patterns among the experimental groups is presented in Table 4. No statistically significant association was observed between experimental group and failure pattern according to the Fisher–Freeman–Halton exact test (*p* = 0.582).

Although numerical differences in failure distribution were observed among groups, these differences were not statistically significant.

### 3.3. Finite Element Analysis

Finite element analysis showed stress concentration predominantly in the cervical region of the root and in the coronal portion subjected to oblique loading in all experimental models. The highest stress concentrations were observed near the interface between the coronal reconstruction and the remaining root structure and along the internal cervical wall of the root canal. No inferential statistical analysis was performed on the FEA outputs because these values represent deterministic numerical solutions generated under predefined model geometries, material properties, boundary conditions, and loading conditions rather than independent experimental observations. Therefore, the FEA results were interpreted descriptively and comparatively.

Among the post-restored models, G5 showed the highest von Mises equivalent stress and maximum principal stress in root dentin. Within the Splendor-SAP models, G6 showed lower root maximum principal stress and slightly lower total deformation than G5. Within the Exacto models, G4 showed slightly lower root maximum principal stress and total deformation than G3.

**Figure 8:**
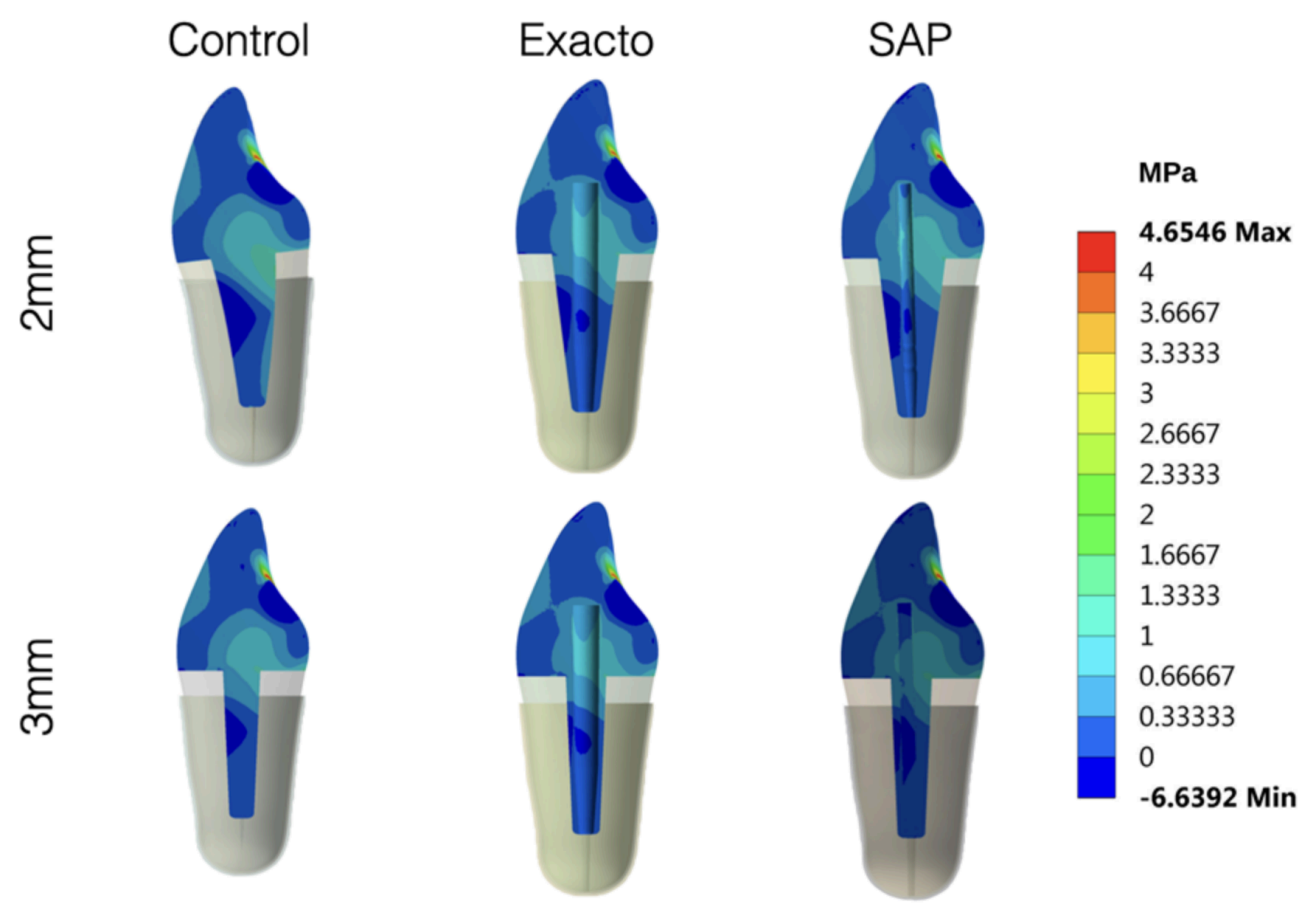
Maximum principal stress distribution (MPa) in the post-and-core build-up for the control, Exacto, and Splendor-SAP models with 2 and 3 mm of remaining root dentin.

**Figure 9:**
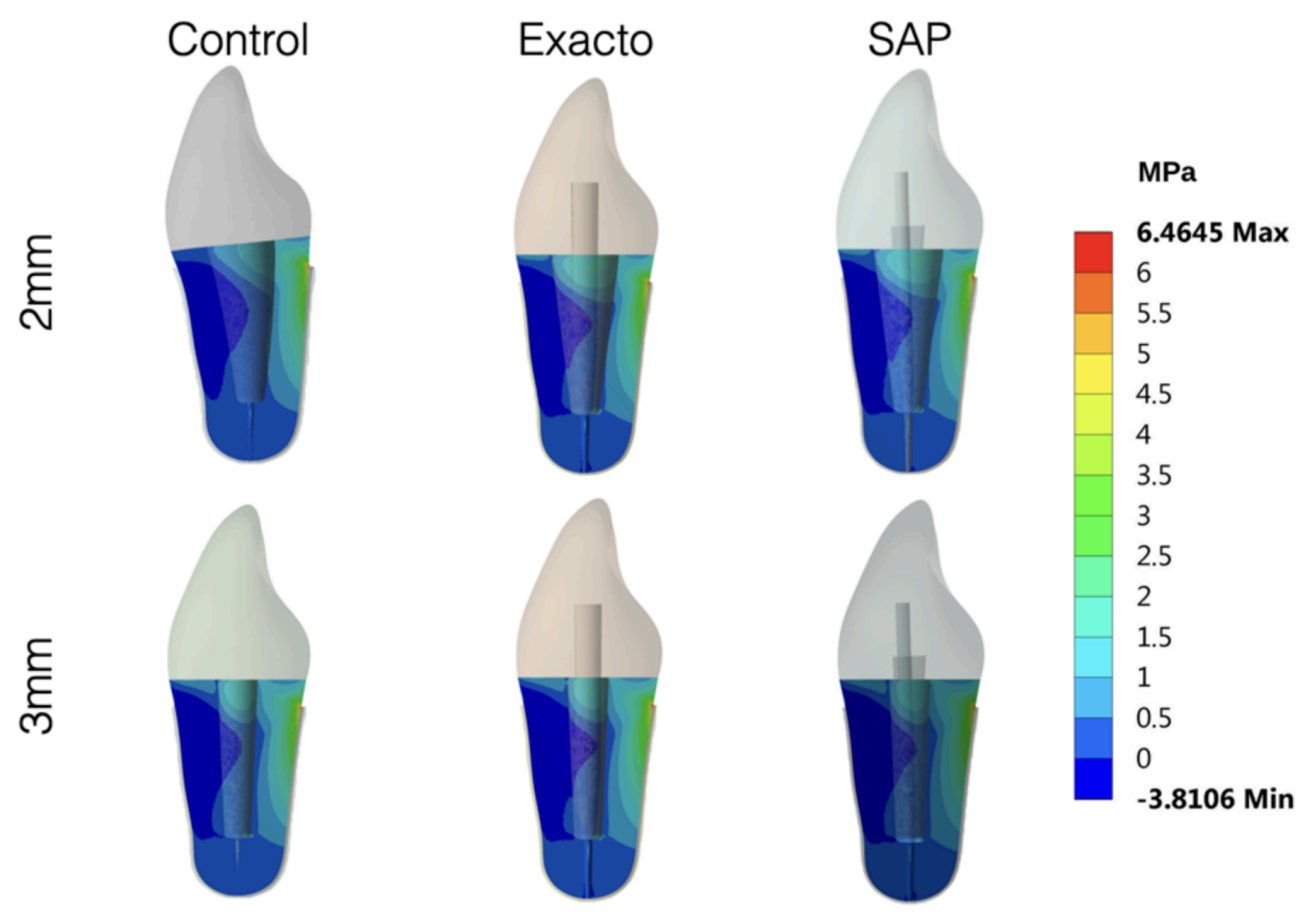
Tensile stress distribution (MPa) in root dentin for the control, Exacto, and Splendor-SAP models with 2 and 3 mm of remaining root dentin.

Overall, relatively similar stress and deformation magnitudes were observed among several experimental configurations, although differences in the magnitude and location of stress concentrations were present. The maximum principal stress distributions in the post-and-core build-up and root dentin are shown in Figure 8 and Figure 9, respectively. The numerical finite element analysis results are summarized in Table 5.

## 4. Discussion

In the present study, glass fiber posts significantly increased the fracture resistance of weakened roots compared with groups without intraradicular posts. The non-reinforced groups G1 and G2 showed the lowest mean fracture resistance values (88.7 N and 95.6 N, respectively), whereas all groups restored with glass fiber posts showed substantially higher values. This finding may be related to the presence of an intraradicular retainer, which may improve the retention and stability of the coronal reconstruction under oblique loading. Nevertheless, intraradicular posts should not be interpreted exclusively as root-reinforcement devices, since their primary clinical role is to provide retention for the core and coronal restoration when insufficient tooth structure remains [20,21,22,23].

Among the post-restored groups, G3 and G5, both with 2 mm of remaining root dentin, showed statistically similar intermediate fracture resistance values (670.9 N and 691.9 N, respectively), whereas G4 and G6, both with 3 mm of remaining root dentin, showed the highest and statistically similar values (786.2 N and 784.8 N, respectively). Thus, no significant difference was observed between the Exacto and Splendor-SAP systems when compared under the same remaining-dentin condition. Instead, among the post-restored specimens, greater remaining root dentin thickness was associated with higher fracture resistance. These findings reinforce the importance of preserving radicular dentin in the mechanical behavior of structurally weakened roots [31,32].

From a biomechanical perspective, glass fiber posts present physical and mechanical characteristics that differ from those of metallic post systems. Their elastic modulus is closer to that of dentin, which may favor more homogeneous stress transfer within the restored tooth and reduce stress concentrations associated with highly rigid intraradicular materials [33]. In addition, adhesive cementation promotes mechanical integration among radicular dentin, resin cement, the glass fiber post, and the core build-up. These characteristics may have contributed to the markedly higher fracture loads observed in the post-restored groups compared with the non-reinforced specimens. However, the present findings should be interpreted as an increase in the resistance of the restored root–core assembly under the experimental loading conditions rather than as evidence that glass fiber posts intrinsically reinforce radicular dentin [20,21,22,23].

The use of bovine teeth in this study allowed better standardization of the specimens and simulation of enlarged canals, compatible with experimental conditions involving weakened roots. Dimensional variability was minimized by selecting straight roots with completely closed apices and by standardizing cervical root diameter and root length before specimen preparation. Nevertheless, biological variability cannot be completely eliminated despite dimensional standardization, and structural differences between bovine and human teeth should be considered [34,35,36,37]. In addition, information regarding animal age was not available, and the initial mechanical characteristics of individual roots were not assessed. These factors represent limitations of the experimental model and should be considered when extrapolating the present findings to clinical conditions.

To simulate a condition of severe root weakening, the crowns were sectioned below the cementoenamel junction, eliminating the remaining coronal structure. This approach allowed evaluation of the behavior of intraradicular systems under a critical condition without a coronal ferrule effect. Although the ferrule effect is recognized as an important factor for the prognosis of endodontically treated teeth, its absence in this study was intentional to create an unfavorable and standardized condition for comparison among groups [4].

Canal preparation was performed to a depth of 12 mm, maintaining 4 mm of filling material in the apical third. This configuration allowed standardized weakening of the cervical and middle thirds of the root while maintaining the apical filling material. Internal enlargement was performed until remaining root dentin thicknesses of 2 and 3 mm were obtained, representing two standardized experimental levels of structural loss rather than clinical threshold values. Among the post-restored groups, specimens with 3 mm of remaining root dentin showed significantly higher fracture resistance than the corresponding groups with 2 mm, supporting the importance of preserving radicular dentin in structurally weakened roots [4].

The fracture resistance test was performed with load application at 45°, reproducing an oblique loading condition commonly used in laboratory studies of anterior teeth [4,38,39]. This loading configuration generates bending and tensile stresses in the root–crown assembly and favors stress concentration in the cervical region of the root. A crosshead speed of 1 mm/min was used to provide progressive load application until fracture and avoid sudden impact on the specimens [4,38,39].

Failure-pattern analysis showed numerical differences among the experimental groups; however, no statistically significant association between experimental group and failure pattern was detected. The non-reinforced groups G1 and G2 showed predominantly type I and type II failures, with no type III fractures. Nevertheless, because these groups failed under substantially lower loads, the absence of catastrophic fractures should not be interpreted as evidence of more favorable biomechanical behavior. Failure in these specimens may have occurred before higher loads could be transferred to the remaining root structure.

Among the post-restored groups, G3 showed the highest numerical occurrence of type III fractures, corresponding to 30% of the specimens. This group had 2 mm of remaining root dentin and was restored with an Exacto post. Although the amount of remaining dentin and the restorative configuration may influence the mechanical response of weakened roots, the present study did not demonstrate a statistically significant association between restorative condition and failure pattern. Therefore, the greater numerical occurrence of type III fractures in G3 should be interpreted cautiously and cannot be independently attributed to the post system or remaining dentin thickness.

Groups G4, G5, and G6 predominantly showed type I and type II failures. Notably, no type III fractures were observed in G6; however, this numerical finding should not be interpreted as evidence of a more favorable failure behavior or superiority of the Splendor-SAP system because the overall distribution of failure patterns did not differ significantly among groups. From a clinical perspective, the distinction between fractures that allow preservation of the remaining root structure and catastrophic fractures remains relevant, since vertical or oblique fractures extending below the simulated bone crest are generally associated with a less favorable prognosis.

Finite element analysis complemented the mechanical test by allowing visualization of stress distribution in the different models. Overall, all groups showed stress concentration in the cervical region of the root and in the coronal portion subjected to oblique loading. This pattern is expected under inclined loading because bending of the post-and-core build-up promotes stress development in the cervical region, which is considered critical for the initiation and propagation of root failures.

In the Exacto groups, increasing the remaining root dentin thickness from 2 to 3 mm was associated with a slight reduction in maximum principal stress in root dentin and total deformation. G3 showed a root maximum principal stress of 6.588 MPa and total deformation of 0.012824 mm, whereas G4 showed 6.4334 MPa and 0.012668 mm, respectively. Although this numerical trend was consistent with the higher fracture resistance observed in G4, the magnitude of the differences in the finite element models was limited and should not be interpreted as direct evidence of a clinically meaningful biomechanical advantage.

In the groups restored with Splendor-SAP, G5, with 2 mm of remaining root dentin, showed the highest von Mises equivalent stress and maximum principal stress in root dentin among the evaluated models, with a root maximum principal stress of 7.0378 MPa. G6 showed a lower root maximum principal stress of 6.4645 MPa and slightly lower total deformation than G5. However, these differences represent numerical trends under the standardized conditions of the finite element model and should not be independently interpreted as evidence of superior clinical performance. Moreover, the fracture resistance of G5 was statistically similar to that of G3, whereas G6 was statistically similar to G4, further indicating that the FEA findings should be interpreted together with the mechanical results rather than in isolation.

Maximum stress values obtained from finite element analysis should not be considered direct predictors of fracture. FEA allows comparative evaluation of stress distribution under standardized loading conditions but does not reproduce the progressive fracture process or the full complexity of the clinical environment. In addition, all interfaces in the present models were considered perfectly bonded, representing an idealized condition of complete adhesion without interfacial sliding or separation. This assumption allowed standardized comparison of the effects of remaining dentin thickness and post-system configuration; however, actual adhesive interfaces may present gaps, degradation, or debonding that could modify stress transfer. Therefore, the FEA results should be interpreted as comparative biomechanical indicators rather than direct predictors of clinical performance.

Accordingly, the association among fracture resistance testing, failure-pattern analysis, and finite element analysis provided complementary information regarding the mechanical behavior of the evaluated restorative conditions. The fracture resistance test allowed comparison of the overall mechanical performance of the groups, whereas failure-pattern analysis characterized the severity of the observed fractures and FEA identified critical regions of stress concentration.

Before the fracture resistance test, all specimens were subjected to 1,200,000 mechanical cycles under an 88 N load, providing a standardized fatigue challenge before progressive loading to failure. However, no thermocycling was performed. Therefore, the possible effects of repeated temperature changes on the adhesive interfaces, resin cement, and restorative components were not reproduced. Furthermore, the fracture resistance test represents an experimental condition of progressive loading until failure, whereas physiological loading is repetitive and variable in magnitude and direction. Consequently, the fracture loads obtained in this study should not be directly equated with physiological masticatory forces or interpreted as predictors of clinical survival.

Although in vitro studies cannot reproduce all the variability of the oral environment, the combined use of mechanical testing, failure-pattern analysis, and FEA provided complementary information regarding the evaluated restorative conditions. Within the limitations of this experimental model, restoration with glass fiber posts substantially increased the fracture resistance of weakened roots compared with non-reinforced conditions. Among the post-restored groups, specimens with 3 mm of remaining root dentin showed higher fracture resistance than those with 2 mm, whereas no significant difference was detected between the Exacto and Splendor-SAP systems under the same remaining-dentin condition. Failure-pattern differences were descriptive and were not statistically associated with the experimental groups, and the numerical differences observed in the finite element models require cautious interpretation. Overall, the findings reinforce the importance of preserving radicular dentin and do not support the superiority of one glass fiber post system over the other under the conditions evaluated.

## 5. Conclusions

Within the limitations of this in vitro study, the absence of an intraradicular post resulted in lower fracture resistance of weakened bovine roots. Both Exacto and Splendor-SAP glass fiber posts substantially increased fracture resistance compared with the non-reinforced conditions. Among the post-restored groups, specimens with 3 mm of remaining root dentin showed higher fracture resistance than those with 2 mm, whereas no significant difference was observed between the Exacto and Splendor-SAP systems under the same remaining-dentin condition.

Failure-pattern distribution was not significantly associated with the experimental groups. Finite element analysis showed stress concentration predominantly in the cervical region of the root and numerical differences in stress distribution among the evaluated models; however, these differences should be interpreted cautiously and do not support the superiority of one glass fiber post system over the other. Overall, the findings reinforce the importance of preserving radicular dentin when restoring structurally weakened roots.

## Figures and Tables

**Table 1:** Distribution of the experimental groups.

Group	Remaining Root Dentin Thickness	Post System
G1	2 mm	None
G2	3 mm	None
G3	2 mm	Exacto
G4	3 mm	Exacto
G5	2 mm	Splendor-SAP
G6	3 mm	Splendor-SAP

Each group consisted of 10 specimens.

**Table 2:** Material properties used in the finite element models.

Material/Structure	Elastic Modulus	Poisson’s Ratio	Reference
Dentin	18.6 GPa	0.32	[4,27,28]
Enamel	84.1 GPa	0.33	[4,27,28]
Glass fiber post	31.5 GPa	0.30	[4,27,28]
Resin cement/core material	5.6–6.0 GPa	0.30	[4,27,28]

**Table 3:** Fracture resistance of the experimental groups.

Group	Remaining Root Dentin	Post System	Mean (N)	SD (N)	95% CI (N)	Statistical Group
G1	2 mm	None	88.7	27.2	69.2–108.2	A
G2	3 mm	None	95.6	22.4	79.6–111.7	A
G3	2 mm	Exacto	670.9	50.2	635.0–706.8	B
G4	3 mm	Exacto	786.2	29.6	765.0–807.3	C
G5	2 mm	Splendor-SAP	691.9	51.6	655.0–728.8	B
G6	3 mm	Splendor-SAP	784.8	38.8	757.1–812.5	C

Different letters indicate statistically significant differences according to the Games–Howell post hoc test (α = 0.05). CI, confidence interval; SD, sample standard deviation.

**Table 4:** Distribution of failure patterns among the experimental groups.

Failure Pattern	G1	G2	G3	G4	G5	G6
Type I	8 (80%)	6 (60%)	4 (40%)	6 (60%)	5 (50%)	7 (70%)
Type II	2 (20%)	4 (40%)	3 (30%)	3 (30%)	4 (40%)	3 (30%)
Type III	0 (0%)	0 (0%)	3 (30%)	1 (10%)	1 (10%)	0 (0%)

Values are presented as *n* (%). No statistically significant association was observed between experimental group and failure pattern (Fisher–Freeman–Halton exact test, *p* = 0.582).

**Table 5:** Finite element analysis results.

Group	Remaining Dentin	Post System	Max. von Mises Stress (MPa)	Max. Root MPS (MPa)	Max. Post/Core MPS (MPa)	Max. Deformation (mm)
G1	2 mm	None	6.8078	6.1210	4.3457	0.011542
G2	3 mm	None	7.6722	6.4234	4.6546	0.012668
G3	2 mm	Exacto	7.2845	6.5880	4.6846	0.012824
G4	3 mm	Exacto	7.5598	6.4334	4.6546	0.012668
G5	2 mm	Splendor-SAP	7.8007	7.0378	4.6004	0.012815
G6	3 mm	Splendor-SAP	7.3960	6.4645	4.6546	0.012660

MPS, maximum principal stress.

## Data Availability

The original data presented in this study are available from the corresponding authors upon reasonable request. The data are not publicly available because they include extensive raw datasets generated during the mechanical tests and finite element analyses.

## Abbreviations

The following abbreviations are used in this manuscript:

Abbreviation	Definition
FEA	Finite element analysis
MPS	Maximum principal stress
PVC	Polyvinyl chloride
